# Histone deacetylase inhibitors SAHA and sodium butyrate block G1-to-S cell cycle progression in neurosphere formation by adult subventricular cells

**DOI:** 10.1186/1471-2202-12-50

**Published:** 2011-05-26

**Authors:** Qiong Zhou, Clifton L Dalgard, Christopher Wynder, Martin L Doughty

**Affiliations:** 1 Department of Anatomy, Physiology and Genetics, Center for Neuroscience and Regenerative Medicine, Uniformed Services University of the Health Sciences, 4301 Jones Bridge Road, Bethesda, Maryland 20814, USA; 2 Department of Biochemistry, Schulich School of Medicine, University of Western Ontario, 112 Siebens-Drake Research Institute, London, Ontario, Canada

**Keywords:** suberoylanilide hydroxamic acid, vorinostat, sodium butyrate, cyclin-dependant kinase inhibitor, p21 (Cip1/Waf1/Cdkn1a), p27 (Kip1/Cdkn1b), cell cycle, chromatin immunoprecipitation

## Abstract

**Background:**

Histone deacetylases (HDACs) are enzymes that modulate gene expression and cellular processes by deacetylating histones and non-histone proteins. While small molecule inhibitors of HDAC activity (HDACi) are used clinically in the treatment of cancer, pre-clinical treatment models suggest they also exert neuroprotective effects and stimulate neurogenesis in neuropathological conditions. However, the direct effects of HDACi on cell cycle progression and proliferation, two properties required for continued neurogenesis, have not been fully characterized in adult neural stem cells (NSCs). In this study, we examined the effects of two broad class I and class II HDACi on adult mouse NSCs, the hydroxamate-based HDACi suberoylanilide hydroxamic acid (vorinostat, SAHA) and the short chain fatty acid HDACi sodium butyrate.

**Results:**

We show that both HDACi suppress the formation of neurospheres by adult mouse NSCs grown in proliferation culture conditions *in vitro*. DNA synthesis is significantly inhibited in adult mouse NSCs exposed to either SAHA or sodium butyrate and inhibition is associated with an arrest in the G1 phase of the cell cycle. HDACi exposure also resulted in transcriptional changes in adult mouse NSCs. Cdk inhibitor genes p21 and p27 transcript levels are increased and associated with elevated H3K9 acetylation levels at proximal promoter regions of *p21 *and *p27*. mRNA levels for notch effector Hes genes and Spry-box stem cell transcription factors are downregulated, whereas pro-neural transcription factors Neurog1 and Neurod1 are upregulated. Lastly, we show HDAC inhibition under proliferation culture conditions leads to long-term changes in cell fate in adult mouse NSCs induced to differentiate *in vitro*.

**Conclusion:**

SAHA and sodium butyrate directly regulate cdk inhibitor transcription to control cell cycle progression in adult mouse NSCs. HDAC inhibition results in G1 arrest in adult mouse NSCs and transcriptional changes associated with activation of neuronal lineage commitment programs and a reduction of stem/progenitor state. Changes in differentiated cell state in adult mouse NSCs treated with HDACi under proliferation culture conditions suggests an intrinsic relationship between multipotency, cell cycle progression and HDAC activity in these cells.

## Background

Adult neural stem cell (NSC) maintenance and differentiation is controlled by intrinsic and extrinsic factors. Many developmental cues have been shown to operate in the adult NSC niche including Wnt [1], sonic hedgehog [2,3], bone morphogenic protein [4] and notch signaling [5,6]. More recently the modification of histone proteins has been identified as an epigenetic regulator of adult neurogenesis [7-9]. Gene expression is epigenetically regulated by enzymatic modifications of histone proteins and changes in histone acetylation by the opposing activities of histone acetyltransferases (HATs) and histone deacetylases (HDACs) is considered the more dynamic form of regulation. HDACs catalyze the removal of an acetyl moiety from the ε-amino group of target lysine residues in histone proteins (reviewed in Grayson et al 2010) and histone deacetylation leads to a condensed chromatin structure that is primarily associated with the repression of transcription (it should be noted HDACs deacetylate other non-histone proteins such as α-tubulin and β-catenin, see [10]).

The ability to inhibit HDAC activity with small molecule HDAC inhibitors (HDACi) has attracted considerable therapeutic attention. Initial interest focused on the application of HDACi as anti-cancer agents and suberoylanilide hydroxamic acid (vorinostat, SAHA, Zolinza) is the first HDACi approved by the FDA for cancer therapy. More recently, therapeutic interest in HDACi has broadened to non-malignant conditions effecting the nervous system [11]. Pre-clinical treatment models demonstrate HDACi exert neuroprotective effects and stimulate neurogenesis in traumatic brain injury (TBI) and ischemia [12,13], restore learning and memory in TBI and neurodegenerative mice [14,15], enhance neuronal differentiation and synaptic plasticity [16,17] and exert antidepressant-like effects [18]. However these same HDACi have also been reported to both prevent [19] or induce neuronal apoptosis in culture [20,21], a contradiction that is likely the result of differences in neuronal cell type, the culture conditions employed and the type of HDACi molecule tested.

In an effort to determine the cell specific effects of HDACi on adult neurogenesis, we have investigated the effects of the broad class I and class II HDAC inhibitors SAHA (a hydroxamate-based HDACi) and sodium butyrate (a short chain fatty acid) on adult mouse NSC biology *in vitro*. Our data indicate these two HDACi exert similar anti-proliferative effects *in vitro *by blocking G1-to-S phase progression in adult mouse NSCs. G1 arrest is associated with the up-regulation of expression of cyclin-dependant kinase (cdk) inhibitors, the down-regulation of stem/progenitor transcription factors and up-regulation of pro-neural transcription factors in adult NSCs. Chromatin immunoprecipitation (ChIP) confirms HDACi directly regulate cdk inhibitor expression in adult mouse NSCs. Finally, we show HDACi treatment under proliferation culture conditions leads to long-term changes in cell fate in adult NSCs induced to differentiate *in vitro*. Combined these data indicate an intrinsic relationship between multipotency, cell cycle progression and HDAC activity in adult mouse NSCs.

## Results

### SAHA and NaB inhibit neurosphere formation by adult mouse NSCs *in vitro*

We have examined the effects of two HDAC inhibitors, the hydroxamate-based HDAC inhibitor suberoylanilide hydroxamic acid (vorinostat, SAHA) and the short chain fatty acid sodium butyrate (NaB). SAHA and NaB are broad class I and class II HDAC inhibitors effective in micromolar (μM) and millimolar (mM) ranges respectively.

**Figure 1:**
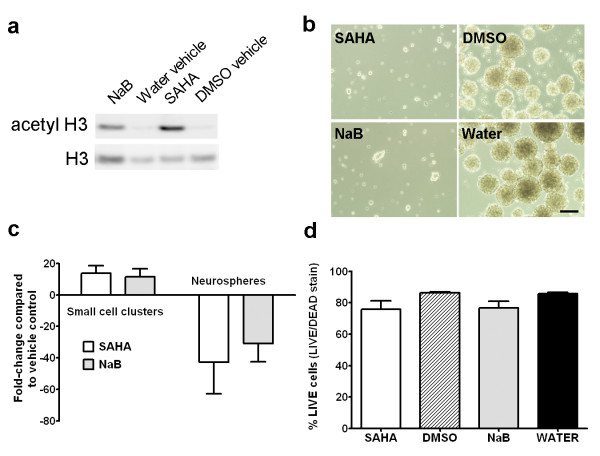
**HDACi treatment results in increased histone H3 acetylation, reduced neurosphere formation and minimal toxicity in adult mouse NSCs grown in proliferation conditions in culture**. (a) Western blot of whole cell extracts demonstrates SAHA and NaB treatment result in increased levels of acetylated histone H3 compared to vehicle controls. (b) Light micrographs of HDACi/vehicle treated cultures illustrate the paucity of neurospheres in HDACi-treated cultures. (c) Quantitative analysis confirms SAHA and NaB inhibit the formation of neurospheres (diameter ≥ 50 μm) and increase the prevalence of small cell clusters (≥ 4 cell, diameter < 50 μm). (d) Flow cytometry using LIVE/DEAD cell stain confirms SAHA and NaB are minimally toxic compared to vehicle controls (n = 3). Scale bar = 100 μm.

We examined the effects of 1 μM SAHA and 1 mM NaB on adult NSCs grown in proliferation culture conditions *in vitro*. Pharmacokinetics indicate 1 μM SAHA treatment falls within the range of clinical use for this compound (effective anti-tumor oral doses of 200-400 mg vorinostat produce peak serum concentrations of 1.1-1.2 μM, see [22]). Western blot analysis of whole cell extracts from adult NSCs treated with HDACi for 48 hours confirms SAHA and NaB treatment results in an increase in histone 3 (H3) acetylation levels when compared to DMSO and water vehicle controls (Figure 1a).

HDACi treatment was associated with clear differences in adult NSC behavior in culture. HDACi-treated adult NSCs exhibited a static behavior when grown in proliferative conditions compared to vehicle-treated controls and failed to produce neurospheres of any significant size or density in cultures (Figure 1b). Quantitative analysis of adult NSCs after 7-days HDACi treatment confirms SAHA and NaB treatment dramatically inhibits the formation of neurospheres (diameter ≥ 50 μm) and increase the prevalence of small (≥4 cells, diameter < 50 μm) cell clusters in culture (Figure 1c). HDACi treatment resulted in minor cell death in culture that was most apparent during the first 1-2 days of treatment. However, quantitative analysis indicates these toxicity effects were not large-scale - LIVE/DEAD staining and flow cytometry reveals SAHA and NaB exposure results in a 10% reduction in cell viability compared to vehicle controls after 48 hours treatment (Figure 1d), a difference that is not statistically significant (NaB vs. vehicle p = 0.10, SAHA vs. vehicle p = 0.12, t-tests, n = 3). Rather, the increased prevalence of small cell clusters suggests HDACi treatment results in a modestly toxic inhibition of the normal proliferative behavior of adult NSCs in our proliferative culture conditions.

### SAHA and NaB block G1- to-S cell cycle progression in adult mouse NSCs *in vitro*

**Figure 2:**
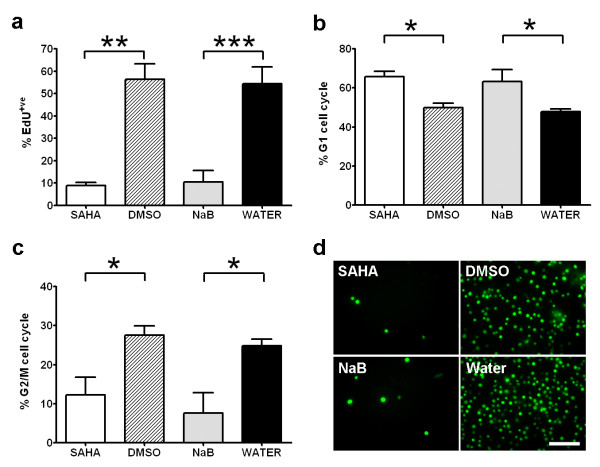
**SAHA and NaB inhibit adult mouse NSC proliferation in culture and arrest cells in G1 phase of the cell cycle**. (a) EdU flow cytometry of live cell-gated adult mouse NSCs demonstrates SAHA (**p < 0.01) and NaB (***p < 0.001) significantly inhibit DNA synthesis. (b) Analysis of DNA content by flow cytometry reveals significant accumulation of adult mouse NSCs in G1phase of the cell cycle following SAHA (*p < 0.05) and NaB (*p < 0.05) treatment. (c) Correspondingly the proportion of cells in G2/M phase is significantly reduced following SAHA (*p < 0.05) and NaB (*p < 0.05) treatment. (d) Fluorescence micrographs of HDACi/vehicle treated adult NSCs pulse-labeled with EdU for flow cytometry. Scale bar = 50 μm. Statistical comparisons performed using one-way ANOVA with post hoc Newman-Keuls multiple comparison tests.

We quantified the effects of HDACi treatment on adult mouse NSC proliferation *in vitro *by comparing the levels of EdU (5-ethynyl-2'-deoxyuridine) incorporation in proliferation culture conditions. EdU is a nucleoside analog to thymidine and is incorporated into DNA during active DNA synthesis. Adult NSCs treated with SAHA or NaB were exposed to EdU (10 μM) overnight (16 hours) following 48 hours HDACi treatment. Cell viability was measured by LIVE/DEAD stain and EdU incorporation compared in live cell gated populations. Flow cytometry measurement of EdU incorporation rates confirmed the addition of either SAHA or NaB significantly inhibited DNA synthesis in adult mouse NSCs *in vitro *(Figure 2a). SAHA treatment resulted in a 6.61-fold reduction (8.9 ± 1.2% versus DMSO vehicle 56.3 ± 7.0%, p < 0.01) and NaB a 5.26-fold reduction (10.4 ± 5.2% versus water vehicle 54.5 ± 7.5%, p < 0.001) in EdU incorporation rates compared to vehicle controls.

Flow cytometry measurement of relative DNA content in combination with EdU incorporation was used to estimate the effects of SAHA and NaB on cell cycle progression in live cells. We used the Dean-Jett-Fox model (Flowjo software) to estimate percentage cell populations in G1 phase and G2/M phase of the cell cycle. This analysis revealed equivalent cell cycle effects in NSCs treated with SAHA or NaB. SAHA or NaB treatment significantly increased the percentage of cells in G1 phase of the cell cycle (SAHA 65.6 ± 2.8% versus 49.9 ± 2.2% DMSO, p < 0.05 and NaB 63.3 ± 6.0% versus 47.5 ± 1.3% water vehicle, p < 0.05), as shown in Figure 2b. Correspondingly, as shown in Figure 2c, SAHA or NaB treatment significantly decreased the percentage of cells in G2/M phase (SAHA 12.3 ± 4.5% versus 27.6 ± 2.4% DMSO, p < 0.05 and NaB 7.7 ± 5.2% versus 24.8 ± 1.7% water vehicle, p < 0.05). Combined these flow cytometry data indicate that the suppression of NSC proliferation by SAHA and NaB results in a G1-to-S phase block in cell cycle progression.

### Gene expression changes induced by SAHA and NaB treatments vary in fold change but not directionality

Genome-wide expression screening indicates HDAC inhibition is associated with expression changes in ≈ 2-5% of the genome [23]. We measured mRNA levels of a cohort of 18 genes implicated in cell cycle progression, stem cell maintenance and NSC fate using qRT-PCR. We harvested RNA for analysis after 48 hours treatment of NSCs in proliferation culture conditions with HDACi or vehicle. Our analysis revealed widespread changes in gene expression following HDACi exposure (Table1). Eight out of 18 genes analyzed showed increased expression and 10 decreased expression when compared to vehicle control. SAHA and NaB treatment induced greater than 2 fold expression changes in a majority of the genes tested (in both a positive (+) and negative (-) direction from vehicle controls). Noticeably, the directionality (+/-) fold change of gene expression changes was consistent between the two HDACi treatments when compared to vehicle controls. We speculate this reflects the similar treatment outcome, G1 arrest, of SAHA and NaB exposure on adult NSCs.

### Gene expression changes induced by SAHA and NaB are consistent with G1 arrest, a reduction in stem/progenitor state and activation of neuronal lineage commitment programs

Gene expression changes in HDACi treated adult NSCs are consistent with the inhibition of G1-to-S phase cell cycle progression. SAHA and NaB treatment result in increased transcription of cyclin dependant kinase inhibitors p21, p27 and p57, and the down-regulation of cyclin dependant kinases Cdk2 and Cdk4 (Table 1). Progression through G1 and S phase of the cell cycle is dependent on Cdk2 and Cdk4 and the activity of these proteins is inhibited by binding of Cdk inhibitors p21, p27 and p57. We also examined genes with functions associated with stem/progenitor or neuronal cell fate. Our analysis revealed SAHA and NaB treatment results in the down-regulation of transcription factors associated with the maintenance of a stem/progenitor cell state and up-regulation of pro-neural transcription factors (Table 1). The progenitor cell cycle regulator c-Myc and stem cell maintaining SRY-box factors are downregulated by SAHA and NaB treatment, as are Notch effector bHLH transcription factors Hes1 and Hes5 (Table 1). In contrast, mRNA levels of pro-neural bHLH transcription factors reveal variable HDACi effects on neuronal lineage commitment genes. Neurog1 and Neurod1 are upregulated whereas Ascl1 is downregulated in adult NSCs treated with SAHA or NaB (Table 1). In summary, qRT-PCR expression data from our gene cohort are consistent with G1 arrest accompanied by a reduction of stem/progenitor state and activation of Neurog1/Neurod1 neuronal lineage commitment programs.

**Table 1:** Gene expression changes induced by SAHA or NaB treatment vary in fold change but not (+/-) directionality and are consistent with G1 arrest, suppression of stem/progenitor and activation of neuronal lineage commitment programs

*Genes Upregulated compared to vehicle control*			
**Gene Symbol**	**Name**	**SAHA**	**NaB**
Ctnnb1	Catenin, beta-1	1.31 ± 0.02	1.82 ± 0.34
Gli1	glioma-associated oncogene	5.26 ± 1.90	4.19 ± 2.77
Neurod1	Neurogenic differentiation1	4.28 ± 1.78	1.45 ± 0.60
Neurog1	Neurogenin 1	9.86 ± 4.35	2.79 ± 1.38
Tcf4	Transcription factor 4	1.50 ± 0.34	1.09 ± 0.18
p21 (Cdkn1a)	Cyclin-dependant kinase inhibitor 1a	5.48 ± 2.98	3.35 ± 0.99
p27 (Cdkn1b)	Cyclin-dependant kinase inhibitor 1b	2.70 ± 0.64	2.34 ± 0.31
p57 (Cdkn1c)	Cyclin-dependant kinase inhibitor 1c	1.89 ± 0.48	3.33 ± 1.17
Shh	Sonic hedgehog	41.57 ± 6.98	6.31 ± 2.37
***Genes Downregulated compared to vehicle control***			
**Gene Symbol**	**Name**	**SAHA**	**NaB**
Ascl1	Achaete-scute complex homolog 1	-2.36 ± 0.71	-3.43 ± 0.92
Cdk2	Cyclin-dependant kinase 2	-4.64 ± 1.91	-8.37 ± 5.87
Cdk4	Cyclin-dependant kinase 4	-3.35 ± 0.70	-4.18 ± 0.17
Ccnd1	Cyclin D1	-1.55 ± 0.63	-1.87 ± 0.17
Hes1	Hairy/enhancer of split, Drosophila homolog of, 1	-3.89 ± 1.90	-5.56 ± 0.57
Hes5	Hairy/enhancer of split, Drosophila homolog of, 1	-11.87 ± 1.18	-23.85 ± 3.15
c-Myc	V-myc avian myelocytomatosis viral oncogene homolog	-1.92 ± 0.77	-2.44 ± 1.05
Olig2	Oligodendrocyte lineage transcription factor 2	-1.06 ± 0.03	-2.83 ± 1.71
Pax6	Paired box gene 6	-2.04 ± 1.18	-4.22 ± 1.92
Sox1	SRY-box 1	-4.46 ± 3.33	-4.96 ± 2.52
Sox2	SRY-box 2	-3.08 ± 1.60	-3.44 ± 1.06
Sox9	SRY-box 9	-2.35 ± 0.25	-1.24 ± 1.06

+/- fold-changes in gene expression in adult mouse NSCs treated with SAHA or NaB were calculated from vehicle controls from ≥ 3 independent qRT-PCR trials

### Increased transcription of Cdk inhibitors is associated with increased histone acetylation at the proximal promoter in HDACi treated adult mouse NSCs

**Figure 3:**
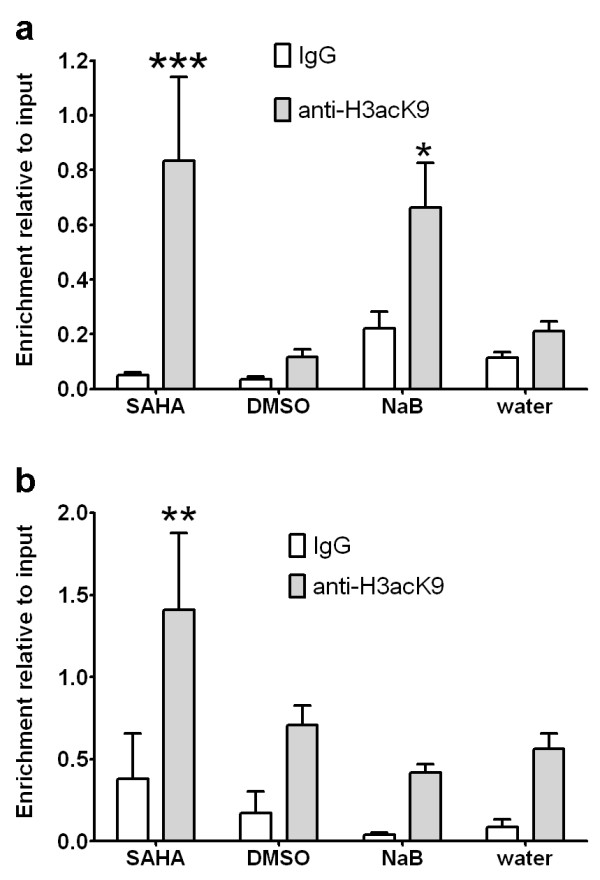
**Upregulated p21 and p27 expression in adult mouse NSCs treated with HDACi is associated with increased acetylation at the proximal promoter**. (a) Chromatin immunoprecipitation (ChIP) reveals significant increases in histone H3 lysine 9 acetylation levels at the proximal promoter of *p21 *in SAHA (***p < 0.001) and NaB (*p < 0.05) treated adult mouse NSCs. (b) ChIP reveals significant increases in histone H3 lysine 9 acetylation levels at the proximal promoter of *p27 *in SAHA (**p < 0.01) but not NaB treated adult mouse NSCs. Statistical comparisons performed using two-way ANOVA with Bonferroni post-tests.

Transcription of the cell cycle regulator p21 is directly regulated by HDAC1 and HDAC2 [24-27] and is re-activated by HDAC inhibitors in tumor cells [28]. To test whether HDAC inhibitor treatment increased histone acetylation levels at the proximal promoter of the *p21 *gene (*Cdkn1a*) in adults NSCs, we performed chromatin immunoprecipitation (ChIP) using antibodies to acetylated lysine 9 on histone H3 (anti-H3acK9). qPCR analysis reveals SAHA induces a 7.2-fold increase and NaB a 3.1-fold increase in H3K9 acetylation levels at the *p21 *promoter compared to DMSO and water vehicle controls respectively (Figure 3a). Statistical analysis confirms a significant increase in H3K9 acetylation in adult NSCs in response to SAHA (p < 0.01) and NaB (p < 0.05), as well as significant interaction between the immunoprecipitation (IP) antisera (p < 0.0001) and treatment (p < 0.05). We also analyzed H3K9 acetylation levels at the proximal promoter of *p27 *(*Cdkn1b*). Expression of p27 is increased in some but not all tumor cells by HDACi and is upregulated in human mesenchymal stem cells by SAHA treatment [29]. As shown in figure 3b, ChIP revealed a 2-fold increase H3K9 acetylation levels at the *p27 *promoter in response to SAHA treatment that was statistically significant (p < 0.05). In contrast, no increase in H3K9 acetylation was observed at the *p27 *promoter in response to NaB treatment (Figure 3b). Combined these data indicate transcriptional activation of p21 and p27 by SAHA is associated with increased H3K9 acetylation at proximal promoter regions, suggesting direct activation of these gene targets. Increased p21 expression and increased in H3K9 acetylation at the *p21 *promoter in NaB treated cells suggests NaB similarly directly activates p21 transcription in adult NSCs. In contrast, the absence of significant changes in H3K9 acetylation at the *p27 *promoter suggests the involvement of alternative mechanisms, possibly acetylation of non-histone proteins, in NaB-mediated increases in p27 expression in adult NSCs.

### Treatment of proliferating adult mouse NSCs with HDACi leads to changes in differentiated cell fate

**Figure 4:**
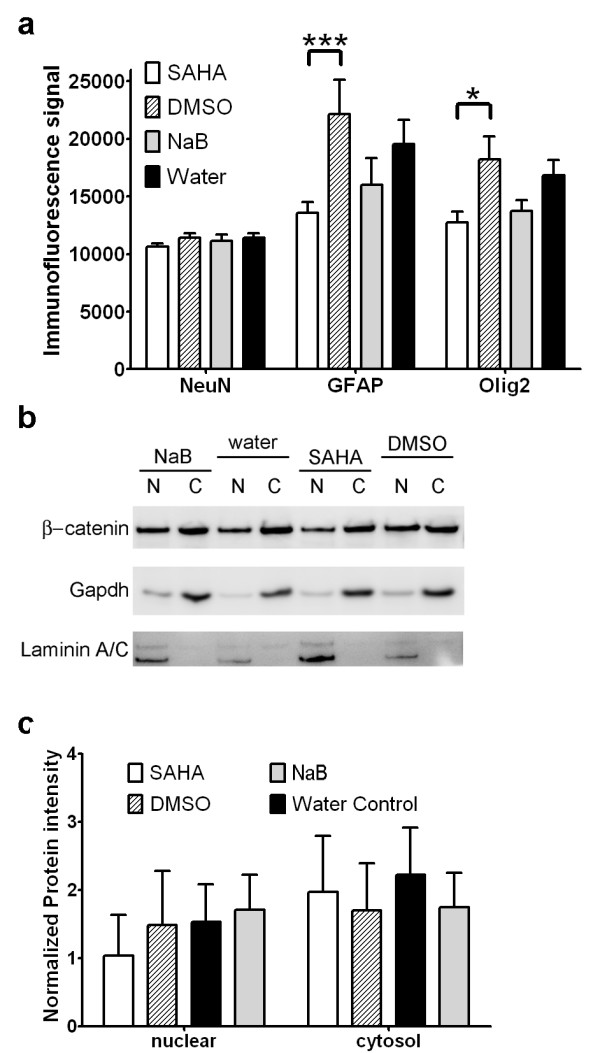
**HDACi treatment under proliferation culture conditions leads to long-term changes in differentiated cell fate**. (a) Immunofluorescence labeling of NeuN, GFAP and Olig2 cell fate markers reveals reduced expression of GFAP (*p < 0.001) and Olig2 (*p < 0.05) in adult mouse NSCs treated with SAHA for 48 hours under proliferation culture conditions and then induced to differentiate for 14 days in culture (two-way ANOVA with Bonferroni post-tests). (b) Western blot of β-catenin protein in nuclear and cytoplasmic fractions of adult mouse NSCs treated with HDACi/vehicle control under proliferation culture conditions. Gapdh and lamin A/C blots are included as loading controls. (c) SAHA and NaB do not significantly alter nuclear/cytoplasmic levels of β-catenin in adult mouse NSCs. Histogram of β-catenin Western blot signal intensities normalized to loading controls (n = 4).

Inhibition of histone deacetylases promotes the acquisition of neuronal and suppresses glial cell fates in differentiating adult NSCs [17,30]. Based on these observations, we predicted HDACi treatment of proliferating adult mouse NSCs would lead to changes in cell fate in adult NSCs induced to differentiate in culture. To test this hypothesis, we developed a 96-well plate immunofluorescence cell fate assay using antibodies to GFAP, Olig2 and NeuN as general markers of astrocytic, oligodendrocyte and neuronal cell fates. Adult NSCs were first treated with HDACi for 48 hours under proliferation culture conditions and then cultured for 14 days under differentiation conditions without HDACi. These assays revealed SAHA significantly reduced glial (p < 0.001) and oligodendrocyte (p < 0.05) differentiated cell fate in culture when compared to DMSO vehicle controls (Figure 4a). However NeuN immunoassays indicate this did not lead to a compensatory increase in neuronal cell fate (Figure 4a). Although NaB treatment similarly reduces the expression of glial and oligodendrocyte cell markers, these differences were not significant compared to vehicle controls. As was the case with SAHA, NaB treatment did not alter NeuN expression levels (Figure 4a).

In an effort to identify molecular mechanisms responsible for SAHA-induced suppression of oligodendrocyte cell fate, we measured β-catenin protein levels in HDACi-treated adult NSCs by Western blot. Combined deletion of *Hdac1 and Hdac2 *in mice is reported to inhibit oligodendrocyte differentiation through the stabilization and nuclear translocation of β-catenin, which in turn represses Olig2 expression [31]. Hence our rationale was that SAHA inhibits Hdac1/2 in proliferating adult NSCs leading to increased nuclear localization of β-catenin and longer-term suppression of oligodendrocyte fates. However, Western blots (n = 4) of adult NSCs treated with HDACi under proliferation conditions do not reveal significant changes in β-catenin nuclear localization in treated cells (Figure 4b and 4c). Fold-changes of β-catenin nuclear protein levels normalized to laminin A/C reveal marginal opposing +/- effects of SAHA (-1.49 ± 0.11) and NaB (1.36 ± 0.33) treatment, indicating SAHA modulates cell fate via mechanisms independent of increased β-catenin stabilization.

## Discussion

In this study we demonstrate the broad class I and class II histone deacetylase inhibitors SAHA and NaB block adult NSC proliferation *in vitro *by blocking G1-to-S progression. HDACi induced cell cycle blockade is accompanied by transcriptional changes consistent with G1 arrest, a reduction of stem/progenitor cell state and activation of neuronal lineage commitment programs in adult NSCs. Furthermore, HDACi treatment of adult NSCs in proliferation culture conditions leads to longer-term changes in cell fate when cells are induced to differentiate in culture.

### SAHA and NaB block G1-to-S cell cycle progression in adult NSCs and activate cdk inhibitor expression

We have shown SAHA and NaB treatment inhibits adult NSC proliferation *in vitro *by arresting cells in G1 phase of the cell cycle. G1 arrest induced by SAHA or NaB treatment has been reported in fibroblasts [32], vascular smooth muscle cells [33] and numerous tumor cell types (see [28]). In many of these contexts, G1 arrest is associated with increased expression of p21 [34] indicating the anti-proliferative effects of SAHA and NaB are, in part, mediated by changes in the expression of cyclin dependant kinase inhibitors.

The functional link between HDAC-mediated regulation of cyclin dependant kinase inhibitor activity and cell cycle progression is supported by a number of genetic studies targeting HDAC genes in mice. Targeted deletion of *Hdac1 *in mice results in embryonic lethality associated with severe reductions in embryonic stem cell proliferation and increased p21 and p27 expression in *null *mutant embryos [27]. Furthermore, disruption of the *p21 *gene (*Cdkn1a*) rescues the proliferation phenotype of *Hdac1-/- *mouse embryonic stem cells (but not the embryonic lethality of the mutation) and chromatin immunoprecipitations confirm the presence of HDAC1 at the *p21 *promoter [26]. Similarly, combined deletion of *HDAC1 *and *HDAC2 *in primary fibroblasts and B-cells results in a strong G1 cell cycle block that is accompanied by increased p21 and p57 transcription [25], and loss of HDAC1/2 catalytic activity results in G1 arrest and up-regulation of p21 in primary and oncogenic-transformed embryonic fibroblasts [24]. Taken together these studies indicate HDAC1 and HDAC2 regulate G1-to-S cell cycle progression in multiple cell types by normally repressing the expression of cyclin dependant kinase inhibitor genes, in particular through transcriptional repression of p21.

In agreement with this, our data reveal SAHA and NaB upregulated p21 mRNA expression in adult mouse NSCs and that transcriptional activation is associated with increased H3K9 acetylation at the proximal promoter region of the p21 gene. This data indicates SAHA and NaB directly increases the acetylation of associated chromatin histone residues to upregulate p21 transcription in adult NSCs *in vitro*, a finding that implies class I and/or class II HDAC activity directly represses p21 gene transcription in adult NSCs to regulate cell proliferation. Similarly our data demonstrates SAHA and NaB upregulated p27 mRNA expression in adult NSCs. However, p27 transcriptional activation is associated with increased H3K9 acetylation at the gene's proximal promoter region of SAHA but not NaB treated adult NSCs. This suggests HDAC activity inhibited by SAHA but not NaB directly represses p27 transcription in adult NSCs to regulate cell proliferation. The fact that p27 mRNA levels are upregulated by NaB treatment irrespective of H3K9 acetylation changes suggests indirect NaB effects on p27 transcription in adult NSCs. We speculate that one of the various acetylated non-histone proteins such as p53 may provide the linkage between HDACi and p27 repression [34,35].

### SAHA and NaB treatment suppresses stem/progenitor and activates neuronal lineage commitment programs in adult NSCs

Our expression data demonstrates SAHA and NaB treatment results in significant changes in gene transcription, changes that differed in magnitude but not directionality from vehicle controls reflecting the similar treatment outcomes of the two HDACi. The three signaling pathways represented within our gene cohort, Sonic Hedgehog [2,36], Wnt/β-catenin [1] and Notch [37] signaling are known to combine to regulate NSC proliferation and neurogenesis in adult rodents. Thus the widespread changes in gene expression indicate HDAC inhibitors alter the output of each of the major regulatory pathways identified in adult NSCs. And since these pathways also control subsequent cell fate selection, it was not surprising that we observed alterations in cell markers such as Olig2 when cells were induced to differentiate.

Overall, the expression data demonstrated HDACi treatment downregulates transcription factors implicated in the maintenance of stem/progenitor cell states and upregulates transcription factors that drive neuronal lineage commitment and differentiation. However, there are a few exceptions that merit discussion. These are the downregulation of pro-neural bHLH factor Ascl1 and the upregulation of Shh and Gli1 mRNAs. Ascl1 downregulation could result indirectly through the modulation of upstream activators such as Tlx [38]. ChIP data suggests direct HDACi effects at the *Shh *loci account for the increase in transcription of Shh and its target Gli1 (data not shown). The fact that Shh-Gli1 upregulation is accompanied by G1 arrest in our study suggests the mitogenic effects of Shh in adult NSCs [3] require normal HDAC function.

In addition to activating cell lineage commitment programs [28,39], HDACi also promote the re-setting of multipotency in terminally differentiated cell lineages by increasing induced pluripotent stem cell (iPS) re-programming efficiency [40-42]. Somatic cell re-programming reactivates expression of Nanog, a homeobox transcription factor necessary for maintaining multipotency in embryonic stem (ES) cells [43]. In ES cells, mSin3A-HDAC complex at the *Nanog *promoter acts to positively regulate Nanog expression under proliferating conditions and HDAC inhibition by TSA downregulates Nanog expression [44], a finding that is analogous to SAHA and NaB repression of multipotent factors in our adult mouse NSC cultures.

In our study both SAHA and NaB downregulate the expression of the stem cell maintaining factor Sox2 in adult mouse NSCs. Conditional gene deletion in mice reveals Sox2 is negatively regulated by Hdac2 in adult neuroblasts and that Sox2 repression is necessary for adult neurogenesis - *Hdac2-/- *neuroblasts ectopically maintain Sox2 expression, fail to mature into neurons and ultimately die of apoptosis [8]. Taking our data into consideration, HDAC regulation of Sox2 expression in adult NSC lineage progression appears to be biphasic: in the first phase, class I and/or class II HDAC activity is required to maintain Sox2 expression and the maintenance of stem/progenitor programs in self-renewing NSCs; in the second phase, HDAC2 functions to downregulate Sox2 in neuroblasts and permit the full activation of neuronal differentiation programs.

### SAHA treatment leads to cell fate changes in differentiated adult NSCs

Our cell fate analysis revealed SAHA treatment of adult NSCs under proliferation culture conditions lead to the long-term suppression of glial and oligodendrocyte cell fate markers GFAP and Olig2 in cells induced to differentiate. Using *Olig1-Cre *and *floxed Hdac1 and Hdac2 *mice, Ye et al. (2009) showed combined deletion of *Hdac1 *and *Hdac2 *in oligodendrocyte progenitor cells inhibited oligodendrocyte differentiation by repressing Olig2 expression. This effect was mediated by the stabilization (reduced phosphorylation) and nuclear translocation of β-catenin, which in turn negatively regulates oligodendrocyte development by repressing Olig2 expression [31]. These authors identified the transcription factor TCF7L2/TCF4 as a co-effector with β-catenin in the regulation of oligodendrocyte differentiation and speculate HDAC1/2 competition with β-catenin for TCF7L2 interaction converts TCF7L2 from a repressor to an activator of oligodendrocyte differentiation. Accordingly, we hypothesized HDACi induced increases in β-catenin stabilization and nuclear localization accounted for Olig2 suppression in our cell fate assays. However Western blot analysis failed to detect significant differences in nuclear protein levels of β-catenin in adult mouse NSCs treated with HDACi under proliferation culture conditions. We speculate HDACi effects on other targets offset this competitive interaction. One possible candidate is Hdac6, a class IIb HDAC. Hdac6 deacetylates β-catenin at lysine 49 (Lys49) to reduce β-catenin phosphorylation and promote β-catenin nuclear localization and c-myc induction [45]. Thus inhibition of Hdac1/2 and Hdac6 activity has the capacity to promote opposing effects on β-catenin stability and nuclear localization by increasing stability (and reducing phosphorylation) via inhibition of Hdac1/2 as well as decreasing stability and nuclear localization as a result of increased Lys49 acetylation and phosphorylation. Indeed differential sensitivities of Hdac1/2 and Hdac6 to SAHA and NaB inhibition might underlie the different +/- fold-changes in β-catenin nuclear localization compared to vehicle controls). The fact that the effects of HDACi are consistent with anti-proliferative responses to pharmacological and genetic interventions targeting the canonical Wnt/β-catenin signaling pathway in adult NSCs [1,46] suggests the net effect of these molecules is to inhibit rather than activate this signaling pathway.

## Conclusion

In summary, the broad class I and class II HDAC inhibitors SAHA and NaB blocked G1-to-S phase progression in proliferating adult NSCs *in vitro*. Gene expression changes induced by SAHA and NaB treatment in adult NSCs vary in fold change but not directionality, consistent with the comparable treatment outcomes of G1 arrest. In addition, the +/- direction of gene changes induced by SAHA and NaB treatment is consistent with G1 arrest accompanied by a reduction of stem/progenitor state and activation of neuronal lineage commitment programs. SAHA and NaB treatment induces increases in the transcription of Cdk inhibitors p21 and p27 in adult NSCs which was associated with elevated H3K9 acetylation levels at proximal promoter regions. This association is consistent with direct SAHA and NaB effects on cell cycle arrest genes in adult NSCs, in common with widely reported HDACi induced growth arrest in normal and transformed cells [28,47]. Finally, we show HDACi treatment under proliferation culture conditions leads to long-term changes in cell fate in adult NSCs induced to differentiate *in vitro*.

## Methods

### Animals

8-10 week old male and female (equal numbers) C57BL/6J mice were purchased from The Jackson Laboratory and housed in the Uniformed Services University's Center for Laboratory Animal Medicine prior to experimental use. Animals were handled in accordance with procedures approved by the Uniformed Services University of the Health Sciences Institutional Animal Care and Use Committee (IACUC). All research complied with DoD regulations as published in DoD Directive 3216.1. The University's Center for Laboratory Animal Medicine is a fully accredited institution with the Association for Assessment and Accreditation of Laboratory Animal Care (AAALAC).

### Neural stem cell culture

Adult neural stem cells (NSCs) were harvested from the subventricular zone (SVZ) of adult C57BL/6J mice for culture. Mice were deeply anesthetized with vaporized isoflurane, decapitated, the whole brain dissected out and the forebrain cut in serial, coronal sections 1 mm thick using a Sorvall Tissue Chopper. The SVZ was dissected out from coronal sections and dissociated to a single cell suspension using Neural Tissue Dissociation kit (Mitenyi Biotech) according to the manufacturer's protocol. Cells were seeded at a cell density of 1 × 10^5 ^cells/ml in mouse NeuroCult^® ^NSC Basal Medium supplemented with mouse NeuroCult^® ^NSC Proliferation Supplement, 20 ng/ml rh EGF, 10 ng/ml of rh FGF-b and 2 ug/ml Heparin (all StemCell Technologies, Vancouver, BC, Canada). Cells were cultured at 37°C, 5% CO_2 _and cultures passaged every 5-7 days. Cells were passaged a minimum of 5 times prior to experimental analysis to ensure a high enrichment of multipotent stem cells. Cells were dissociated to a single cell suspension in 0.025% Trypsin-EDTA (Invitrogen, Carlsbad, CA, USA) and re-seeded in culture medium at 1 × 10^5 ^cells/ml.

### HDAC inhibitor Treatment

HDAC inhibitors (HDACi) suberoylanilide hydroxamic acid (SAHA, Selleck chemicals, Houston, TX, USA) or sodium butyrate (NaB, Sigma, St. Louis, MO, USA) were added to NSC cultures 2 hours post-passage. SAHA or NaB were added to a final concentration in culture media of 1 μM or 1 mM respectively from freshly prepared solutions of 100 μM SAHA/DMSO and 100 mM NaB/water. An equal volume of DMSO or water was added to cultures as vehicle controls.

### Small cell cluster and neurosphere counts

Following 7 days HDACi treatment, adult NSCs were incubated with a 0.2% solution of trypan-blue dye and small cell clusters/neurospheres excluding dye counted. Average small cell clusters/neurospheres were estimated from 10 randomly selected 2.1025 mm^2 ^counting areas using an optical graticule and Zeiss inverted A1 microscope. The criteria for inclusion as small cell clusters were clusters of ≥ 4 cells but a cell aggregate sphere diameter of < 50 μm. The criteria for neurospheres was a cell aggregate sphere of ≥ 50 μm in diameter. Small cell cluster/neurosphere numbers were compared to values obtained from simultaneous vehicle control cultures to calculate fold-changes. Counting area selection and cell counting were independently performed by two "blinded" investigators.

### Flow Cytometry, Click-iT™ 5-ethynyl-2'-deoxyuridine (EdU) and CellCycle DNA labeling

All procedures were performed according to the manufacturer's instructions (Invitrogen). Adult NSCs cultures were pulsed with 10 μM 5-ethynyl-2'-deoxyuridine (EdU) in cell culture media for 4 or 16 hours at 37°C, 5% CO_2_, the cells harvested and labeled with Alexa Fluor^® ^488 dye by Click-iT™ chemistry. For combined cell cycle analysis, cells were co-labeled with CellCycle 488-red (7-AAD) and LIVE/DEAD^® ^Fixable Violet stains. For cells quantified for EdU content only, cells were co-labeled with LIVE/DEAD^® ^Fixable Red stain (Invitrogen).

DNA content and proliferation status data acquisition was performed on a LRS II Flow Cytometer System at low flowrate with standard compensation and double discrimination parameters. Acquired data was analyzed using FlowJo 9.0.1 (Ashland, OR, USA). Briefly, events were first gated based on FSC and SSC parameters for the prominent cell population, then gated based on LIVE/DEAD signal. Live cells were analyzed for EdU incorporation for proliferation and also DNA content. Cell cycle analysis of the live population was performed in FlowJo using a Dean-Jeff-Fox model to compute the percentage of events in each phase.

### Real-time quantitative reverse transcription PCR (qRT-PCR)

First-strand cDNA was synthesized from 1 ug of total RNA using random primer/oligo(dT) primer according to the manufacturer's instructions (ABI, Carlsbad, CA, USA). Synthesized cDNA was diluted to final concentration of 10 ng/ul for qPCR using SYBR Green Master Mix (ABI) on an ABI 7500 Real-Time PCR System. Optimum primers were designed using NCBI's Primer BLAST http://www.ncbi.nlm.nih.gov/tools/primer-blast/. Primer sequence pairs used are listed in table 2. Primer specificity was confirmed by verifying a single PCR product had been generated using UV gel electrophoresis, as well as by confirming the melting temperature of the product had a single value on dissociation plots. Gene of interest (GOI) Ct values were normalized to internal control 18S, Actb and/or Hprt1 to calculate ΔCt. Fold-changes (F) in gene of interest (GOI) expression were estimated using the ΔΔCt method: F = 2^-ΔΔCt ^where ΔΔC_t _= GOI ΔC_t _HDACi - GOI ΔC_t _vehicle control. Ct values were measured in triplicate and recorded if the standard deviation was < 0.3. All values represent a minimum of 3 biological replicates.

**Table 2:** Primer pairs used for qRT-PCR expression analysis

Gene	Forward primer	Reverse primer
Ascl1	AACCGGGTCAAGTTGGTCAA	CGTCTCCACCTTGCTCATCTTC
Ccnd1	AGAGGGCTGTCGGCGCAGTA	GGCTGTGGTCTCGGTTGGGC
Cdk2	TGCTGAAATGGTGACCCGCAG	TGCCGAGCCCACTTGGGGAA
Cdk4	GGTGTATGGCGCCGCAGGAA	GCAGGGGATCTTACGCTCGGC
p21 (Cdkn1a)	TCCAGGAGGCCCGAGAACGG	CTCCGAACGCGCTCCCAGAC
p27 (Cdkn1b)	GCACTGTGGAGCAGACGCCC	TCTGCCAGCAGTCCTGGGGT
p57 (Cdkn1c)	CCAATCAGCCAGCCTTCGACCA	CAGGCGCTGCTACGCGCTAT
Ctnnb1	AGGCGAACGGCATTCTGGGC	ACGCACCGTCCTTCGTGCTG
Gli1	GCTTGGATGAAGGACCTTGTG	GCTGATCCAGCCTAAGGTTCTC
Hes1	TGGCGGCTTCCAAGTGGTGC	GATGACCGGGCCGCTGTGAG
Hes5	GCCTGGAGCGGACCAGAGGA	CAGCCGGAGGAGGGAGCCTT
c-Myc	ACCCGCTCAACGACAGCAGC	CCGTGGGGAGGACTCGGAGG
Neurod1	CCGCCACACGCCTACA	CAAACTCGGCGGATGG
Neurog1	GACCTGCATCTCTGATCTCG	TGTAGCCTGGCACAGTCCTC
Olig2	CTCCGACGCCAAGTGAGCCG	AATCCCCTAGGCCCAGCCCG
Pax6	AAGCAACAGATGGGCG	GCTTCATCCGAGTCTTC
Shh	CACCCCCAATTACAACCCCGACA	TCCAGGCCACTGGTTCATCACAGA
Sox1	CTGGGCGCCCTCGGATCTCT	GGACACGGTGCCCGTGAGTG
Sox2	GAAAGAAAGGAGAGAAGTTTGGAG	ATCTGGCGGAGAATAGTTGGG
Sox9	TCGGTGAAGAACGGACAAGC	TGAGATTGCCCAGAGTGCTCG
Tcf4	TGGAGGCCATCCAAGCCCGT	TGCTGGTGGCAACCCTGAACG

### Chromatin Immunoprecipitation (ChIP)

Adult NSCs were treated for 10 minutes with 1% formaldehyde in PBS at room temperature. The cells were lysed in cell lysis buffer (5 mM PIPES (pH = 8.0), 85 mM KCl, 0.5% NP-40 and 1× protease Inhibitors, Pierce, Rockford, IL, USA) and the crude cell lysate transferred to a sonication buffer (1× PBS, 1% NP-40, 0.5% sodium deoxycholate and 1× protease Inhibitors, Pierce). The lysate was sonicated under conditions yielding fragments ranging from 500 to 1,000 bp. Samples were subsequently precleared at 4°C with recombinant protein G agarose beads (Roche, Indianapolis, IN, USA). Precleared lysate (100 μl) diluted in immunoprecipitation buffer (0.01% SDS/1.1% Triton X-100/1.2 mM EDTA/16.7 mM Tris·HCl, pH 8.0/167 mM NaCl) was used for a 16 hour overnight immunoprecipitation with 5 μg rabbit anti-Histone H3 (acetyl K9) antibody or ChIP rabbit IgG control (both Abcam) at 4°C. Immunoprecipitated complexes were collected by incubation with recombinant protein G agarose beads (Roche) for 1-4 hours at 4°C. After washing and elution, formaldehyde cross-linking was reversed with a 16 hour overnight incubation at 65°C. Samples were purified using PCR purification kit columns (Qiagen, Valencia, CA, USA) and used as a template for qPCR.

Enrichment relative to input was calculated from qPCR values from ChIP templates as follows. Standard curves were generated from serial 10-fold dilutions of 10% of input DNA and plotted on a log10 scale to obtain the linear relationship between Ct value and template concentration. Using this equation, qPCR values were normalized to input and enrichment compared to IgG control samples and differences statistically determined (2-way ANOVA with Bonferroni post-tests). p21 (Cip1/Waf1/Cdkn1a) primers used were GGGTGCAGGGCTGGCTGAAC and TGGACATGGTGCCTGTGGCT, p27 (Kip1/Cdkn1b) primers were CCCAGACCTGCGCGCTACTG and GACCACCGCCTCGCCTCTCT.

### Immunofluorescence Cell Fate Quantification

Passaged adult NSCs were seeded at 2,000 cells/well in ploy-D-lysine coated black frame/white wall 96-well tissue culture plates (Perkin Elmer, Waltham, MA, USA) in NeuroCult^® ^NSC Basal Medium supplemented with mouse NeuroCult^® ^NSC Proliferation Supplement, 20 ng/ml rh EGF, 10 ng/ml of rh FGF-b, 2 ug/ml Heparin (all and SAHA or NAB added to culture medium as described previously. In each 96-well plate, SAHA, NaB, DMSO or water vehicle was added to equal numbers of culture wells. After 48 hours medium was replaced with NeuroCult^® ^NSC Basal Medium supplemented with mouse NeuroCult^® ^NSC Differentiation Supplement. Medium was refreshed after 7 days and cells grown for a total of 14 days in culture before fixation in 4% paraformaldheyde in 1× PBS. Plates were washed in 1× PBS and incubated with primary antibody at 4°C overnight. Each plate was incubated with a single primary antibody diluted in 1× PBS containing 1% NGS, 0.2% Triton X-100. The primary antibodies and dilutions used were as follows: 1: 500 dilution of mouse monoclonal anti-NeuN (Millipore, Billerica, MA, USA); 1:2,000 dilution of mouse monoclonal anti-GFAP (Abcam, Cambridge, MA, USA); 1: 1,000 dilution of rabbit polyclonal anti-Olig2 (Millipore). The following day plates were washed in 1× PBS and incubated with Alexa Fluor^® ^488 conjugated goat ant-mouse IgG or Alexa Fluor^® ^555 conjugated goat anti-rabbit IgG secondary antibodies (Invitrogen) at 1:1,000 dilution in 1× PBS for 60 minutes at room temp. Plates were then washed in 1× PBS and the fluorescence signal in each well measured using a FLUOStar Optima microplate reader (BMG FLUOStar, Cary, NC, USA). The average fluorescence value for each treatment was calculated and data obtained from 6 independent replicates.

### Western blot

Nuclear and cytoplasmic proteins were isolated from adult NSCs using NE-PER^® ^reagents (Thermo Scientific) according to the manufacturer's instructions (Thermo Scientific, Rockford, IL, USA). Protein fractions were stored at -20°C in 1x Halt™ Protease Inhibitor Cocktail (Thermo Scientific) until use. Protein extracts were mixed with reducing sample buffer, separated by SDS-PAGE and electro-transferred to PVDF membrane. 20 μg protein was loaded per lane. All washes and antisera incubations were performed in 5% skim milk in TBS with 0.1% Tween-20. Blots were incubated with primary antisera overnight at 4°C on a rocking platform. Primary antisera and dilutions used were as follows: 1: 2,000 mouse anti-β-catenin (BD Biosciences, Franklin Lakes, NJ, USA); 1: 1,000 mouse anti-Gapdh (Millipore): 1,000 mouse anti-Laminin A/C (BD Biosciences). The following day blots were washed 3 times and incubated with a 1:5,000 dilution of peroxidase-conjugated goat anti-mouse IgG secondary antisera (Cell Signaling Technologies) for 1 hour at room temp. Blots were developed in Immobilon chemiluminescent ECL substrate (Millipore) for 5 minutes at room temp and the fluorescent signal captured using a Fujifilm LAS-3000 phospho-imager (Fujifilm, Valhalla, NY, USA). Immunosignal intensities (protein density) were measured using Fujifilm Multi Gauge software. Images were optimized for brightness and contrast for publication.

## Abbreviations

ChIP: chromatin immunoprecipitation; H3: histone 3; H3acK9: acetylated lysine 9 of histone H3; HDAC: histone deacetylase; HDACi: histone deacetylase inhibitor; NaB: sodium butyrate; NSC: neural stem cell; SAHA: suberoylanilide hydroxamic acid

## Authors' contributions

ZQ performed all the cellular and molecular work reported in this manuscript. CD conducted the flow cytometry, associated data analysis and participated in the design of the study. CW participated in the design of the study, in the optimization of chromatin immunoprecipitation protocols and preparation of the manuscript. MD conceived of the study, participated in its design and coordination and drafted the manuscript. All authors have read and approved the final manuscript.
